# Effect of Papaver rhoeas hydroalcoholic extract on blood corticosterone and psychosocial behaviors in the mice model of predator exposure-induced post-traumatic stress disorder

**DOI:** 10.1016/j.heliyon.2023.e18084

**Published:** 2023-07-07

**Authors:** Shima Shahyad, Hedayat Sahraei, Kamal Mousallo, Gila Pirzad Jahromi, Mohammad Taghi Mohammadi

**Affiliations:** a Neuroscience Research Center, Baqiyatallah University of Medical Sciences, Tehran, Iran; b Department of Physiology and Medical Physics, Faculty of Medicine, Baqiyatallah University of Medical Sciences, Tehran, Iran

**Keywords:** Papaver rhoeas, Corticosterone, Psychosocial behaviors, HPA axis, PTSD

## Abstract

The function of hypothalamic-pituitary-adrenal (HPA) axis and psychosocial behaviors are affected in post-traumatic stress disorder (PTSD). Based on presence of several beneficial alkaloids in Papaver rhoeas (PR) plant, we assessed the effects of PR hydroalcoholic extract on blood corticosterone and psychosocial behaviors in the mice model of predator exposure-induced PTSD. Male NMARI mice were assigned into two main groups (control or PTSD) according to stress exposure (presence or absent of the predator). Each main group was divided into four subgroups according to treatment with the different doses of PR extract. Mice were treated intraperitoneally by PR extract at three different doses (1,5&10 mg/kg) 30 min before the beginning of test on days 1, 2&3. Corticosterone concentration determined in the blood samples on days 1, 3&21, and mice examined for the psychosocial behaviors on the third day. PTSD induction in mice by exposing to hungry predator increased blood corticosterone and changed the psychosocial and physiological behaviors. PR extract decreased blood corticosterone in PTSD mice on the third day as well as 21st day. Also, PR extract improved the psychosocial and physiological behaviors in PTSD mice. Moreover, PR extract increased blood corticosterone in control mice at a dose-response manner. PR extract is able to decrease blood corticosterone in PTSD condition and probably prevent the HPA hyperactivity in PTSD mice when exposed to the stress stimuli. Accordingly, decreased blood corticosterone by PR extract might be involved in improvement of the physiological and psychosocial behaviors in PTSD mice.

## 1 Introduction

Post-traumatic stress disorder (PTSD) is one of the most common psychiatric diseases that characterized by several psychological symptoms after experiencing severe physical or psychosocial traumatic events [[1], [2], [3]]. The most important symptoms of this psychological disorder are anxiety, restlessness, disturbing thoughts and memories of the event, trying to avoid the stimuli associated with the traumatic event, changes in thinking and feelings, and increased fight and flight reactions [[4], [5], [6]]. Learning and memory are other relevant variables that may be affected following occurrence of PTSD [7,8]. These symptoms may persist for more than a month after happening the traumatic events. Many neurotransmitter systems and neurobiological mechanisms are associated with PTSD occurrence [4,9]. In the pathophysiology of PTSD, the early signs may be related to change the structure and functions of different brain parts, including the amygdala, locus coeruleus, hippocampus and the cortical parts of brain as well as noradrenergic system and hypothalamic-pituitary-adrenal (HPA) axis [10,11]. Activation of HPA axis plays a crucial role against the psychophysiological challenges, and accordingly, the plasma levels of cortisol rises in response to the psychosocial stress [12,13]. Interestingly, a change in plasma levels of cortisol and damage to glucocorticoid signaling pathways have been cited as the main causes of the psychosocial disorders [13,14]. Furthermore, a decrease in the plasma, salivary and urinary concentrations of glucocorticoids has been demonstrated in the PTSD conditions by clinical and experimental studies [10,15,16]. Hence, improving the functions of HPA axis as well as correcting blood cortisol levels might be a probable way to prevent the risk of developing PTSD symptoms.

Papaver rhoeas (PR) plant grows in several areas of Western Europe and East Asia as well as in the different regions of Iran [17]. It, as an herbal drug, is traditionally used largely by the people in the mentioned countries as a therapy for coughs and minor sleep disorders as well as for the symptomatic treatment of several psycho-neurological disorders in adults. According to the behavioral and pharmacological findings by Soulimani et al., the ethanolic and aqueous extract of Papaver rhoeas decreased locomotor, exploratory and postural behaviors in mice [18]. Also, the sedative effects of Papaver rhoeas extract have been shown at a 400 mg/kg dosage by previous findings [18]. Using phytochemical analysis, presence of many different types of the beneficial alkaloids have been demonstrated in Papaver rhoeas extract, including rhoeadine, protopine, allotropine, berberine, coulteropine, coptisine, isocorhydine, sinactine, isorhoeadine, roemerine and rhoeagenine [19,20]. Existence of non-alkaloidal secondary metabolites such as anthocyanins has been identified in Papaver rhoeas extract by the analytical studies using high performance liquid chromatography [21]. Papaver rhoeas extract also contains several substances such as papauric acid, papaverine and muconic acid, which can affect the release of different brain neurotransmitters [18,21,22]. According to the reports, the alkaloids of Papaver rhoeas extract have neuroleptic effects and also these compounds behave as the dopaminergic antagonists in the animal studies [23]. *Anti*-glutamate effects of Papaver rhoeas extract is another relevant finding that demonstrated by the experimental studies [24,25]. It is also claimed that Papaver rhoeas extract is useful for many respiratory symptoms such as bronchitis, pneumonia, and rash fever as well as to calm intestinal and urinary irritation [18].

According to the beneficial effects of Papaver rhoeas extract in management of the psychological symptoms and affecting the release of different brain neurotransmitters in the experimental studies, the present study aimed to evaluated the effects of Papaver Rhoeas hydroalcoholic extract on blood corticosterone concentrations and psychosocial behaviors in the mice model of predator exposure-induced PTSD.

## 2 Materials and methods

### 2.1 Animals

Male NMARI mice, weighing 25–30 g, were used to perform the present study. Mice were kept, in pairs, in the standard cages at the animal care center of the Neuroscience Research Center in Baqiyatallah University of Medical Sciences. All ethical considerations regarding to keep, care and work with laboratory animals were considered during the study. Animals received standard mouse chow and tap water during the study except to test day. Mice were kept in a standard situation with controlled temperature and humidity as well as a controlled environment with 12:12 h light/dark cycle.

### 2.2 Preparation of Papaver rhoeas hydroalcoholic extract

To prepare Papaver Rhoeas hydroalcoholic extract, first, the mentioned plant harvested from the farms of Kermanshah (Western Iran). To identify the Papaver Rhoeas plant by an expert, the collected plants transferred to the pharmacognosy laboratory of the Faculty of Pharmacy in Shahid Beheshti University of Medical Sciences. After drying in the shade, the plants (all parts including petal, steam and leaf) were put in an electric grinder and grounded them into a powder. Then, 100 g of the powder was mixed with 500 mL of ethanol (purity of 100%) and 500 mL of distilled water as a solvent and kept in a room temperature for 24 h. During this time, the solution was gently stirred. After that, the supernatant was separated, filtered for several times by a suitable filter and kept in room temperature for 24 h. Finally, the concentrated liquid was transferred to a suitable container (to easily separate) and left for 24 h to evaporate the alcohol and water until a dry extract was obtained. This extract was dissolved in saline and injected at the appropriate doses.

### 2.3 Drugs

Hydroalcoholic extract of Papaver Rhoeas was used to treat the animals in the current study. The prepared dry extract of Papaver Rhoeas was dissolved in distilled water. Mice were administered intraperitoneally by the solution of extract at three doses of 1 mg/kg, 5 mg/kg and 10 mg/kg 30 min before the beginning of test on days 1, 2 and 3. Also, control animals received intraperitoneally saline in the equal volume of extract solvent as vehicle.

### 2.4 Induction of PTSD

In the present study, exposing to the predator was used to induce PTSD. After a week of adaptation, the mice were separately located in a place to induce PTSD, with size 10 × 20 × 30 cm, which made of transparent plexiglass, and had suitable pores for facilitating the exchange of smell and sound. After 10 min, the mice were exposed to a hungry cat (as predator) for 10 min that habituated to eating mice. Since the cat was hungry, it was attacking the animal many times but was not able to access it. Then, the animals were returned to their cages for continuing the study. This process was repeated on the second and third days. Each time after returning to the place of keeping, the amount of water and food consumption by the animals were recorded. The blood samples were taken from the choroid plexus of animal's eyes to determine the plasma corticosterone on the first day before the exposure and the third day after exposure of mice to the predator. On 21st day, the mice were again placed in the stress box for 10 min, without exposure to the predator. In this way, the animals were only in the place where they were threatened with death. The blood samples were taken from the choroid plexus of animal's eyes on 21st day to determine the plasma corticosterone before and after locating the mice to the stress box without exposure to the predator.

### 2.5 Protocols and grouping

**Fig. 1:**
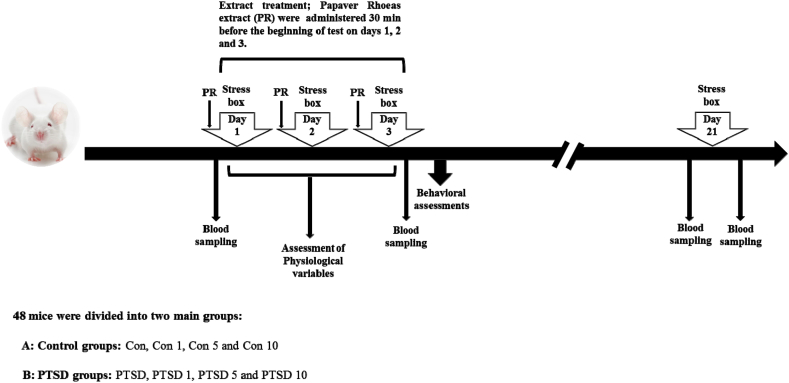
Schematic representation of the experimental design of the study.

To perform the study, the mice (N = 48) were divided into two main groups (control or PTSD groups) according to exposure to stress (presence or absent of predator). Each main group divided into four subgroups according to treatment of animals by saline or the different doses of Papaver rhoeas extract (each subgroup; n = 6). The sample size was confirmed by an expert statistician. The mice of control group (Con) were administered saline as vehicle during the study and placed in stress box without exposure to predator on the days 1, 2, 3 and 21. Treated control groups (Con 1, Con 5 and Con 10) received Papaver Rhoeas extract at different doses (1 mg/kg, 5 mg/kg and 10 mg/kg, respectively) during the test. These mice were located in the stress box without exposure to the predator on the days 1, 2, 3 and 21. The mice of PTSD group (PTSD) were administered saline as vehicle during the study and placed in stress box with exposure to predator on the days 1, 2, 3 and without presence of predator on the day 21. Treated PTSD mice (PTSD 1, PTSD 5 and PTSD 10) received Papaver Rhoeas extract at different doses (1 mg/kg, 5 mg/kg and 10 mg/kg, respectively) during the study. These mice were placed in the stress box with exposing to the predator on the days 1, 2, 3 and without presence of the predator on the day 21 (Fig. 1).

### 2.6 Determination of body weight, food intake, water intake and sugar intake

Body weight of mice (gram) was measured by a digital scale at the beginning of test on the first day and after termination of test on the third day. Consumption of daily food (gram) by the mice was determined via a digital scale on days 1, 2 and 3 of the test. Also, daily water intake by the animals (mL) was determined using a graduated cylinder on days 1, 2 and 3 of the test. Consumption of daily sugar by the animals (gram) was calculated according to the content of solved sugar and measured water intake on days 1, 2 and 3 of the study.

### 2.7 Assessment of psychosocial behaviors

At termination of the test on the third day, the animals were examined for the occurrence of the psychosocial behaviors by the open field test. First, the mice were placed into the center of the open field apparatus, a plexiglass box with dimensions of 10 × 20 × 30 (length, width, height) whose floor was divided into eight equal parts by drawing transverse lines, and the psychosocial and behavioral parameters (locomotor activity, grooming, sniffing and rearing) were recorded over a set time (5 min) by a video camera. These videos were then reviewed and analyzed by a person who was unfamiliar with the animals, and the mentioned behaviors for each animal were counted.

### 2.8 Determination of plasma corticosterone levels

The plasma levels of corticosterone were measured in the blood samples, which taken from the animal's eyes on the day 1 before locating in the stress box, day 3 after locating in the stress box and day 21 both before and after locating in the stress box. After taking the blood samples, plasma was separated by centrifugation. The plasma concentration of corticosterone (ng/mL) was determined by using a corticosterone EIA kit (Zellbio, Germany), according to the manufacturer's instruction. First, the plasma samples (10 μL) were transferred to the ELISA plate and the solution of corticosterone EIA kit mixed with them. After incubation at 37 °C, the absorbance of each sample was read by an ELISA reader at a wave length of 450 nm. Finally, the plasma concentration of corticosterone in each sample was calculated using a standard curve that obtained from the different concentrations of control samples, according to the manufacturer's protocol.

### 2.9 Statistical analysis

In the current study, data analyze was performed using SPSS software (V.21) by an expert statistician. First, the normality of data was done by the Kolmogorov-Smirnov test. ANOVA and Tukey's Post-Hoc test was utilized to analyze the data of between groups at different times. The methods of paired-samples T-test (only for results of animal weight) and repeated measures (for data of food intake, water intake and sugar intake) were used to analyze the data between two different times and three different times in each group, respectively. All states, P values less than 0.05 were expressed as a significant difference.

## 3 Results

### 3.1 Effect of Papaver rhoeas extract on body weight, food intake, water intake and sugar intake

**Fig. 2:**
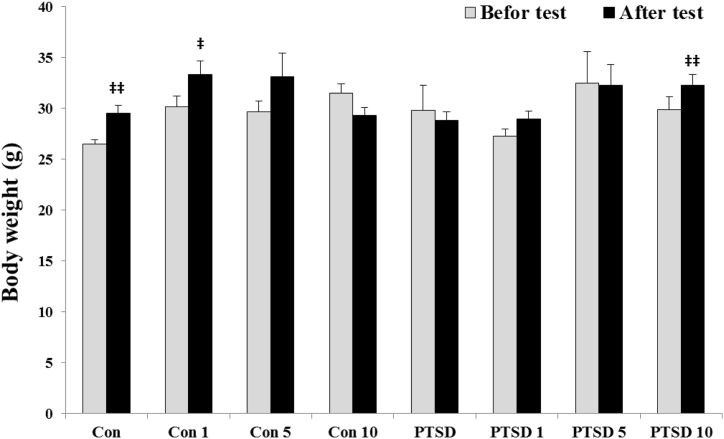
Body weight (g) of mice in control and PTSD groups before placing in the stress box on the day one and after placing in the stress box on the day 3. All values are presented as mean ± SEM. There was no significant difference between groups on the days one and 3. **‡**P < 0.05, and **‡‡**P < 0.01 as significant differences compared to day one for each group.

Alterations of animal body weight are shown at Fig. 2. ANOVA test did not show a significant difference in the body weight of mice between the different groups on the days 1 [F (7, 40) = 1.635] and day 3 [F (7, 40) = 2.277]. T-Test analysis between the days 1 and 3 in each group showed that the animals of control group (p < 0.01) and treated control groups (at doses 1 and 5 mg) had a weight gain during the test. While PTSD mice showed a body weight reduction during the test, the treated PTSD mice by Papaver rhoeas extract did not show a reduction in body weight. In this regard, treated with Papaver rhoeas extract at dose of 10 mg significantly increased body weight of the PTSD mice during the test (p < 0.01).

**Fig. 3:**
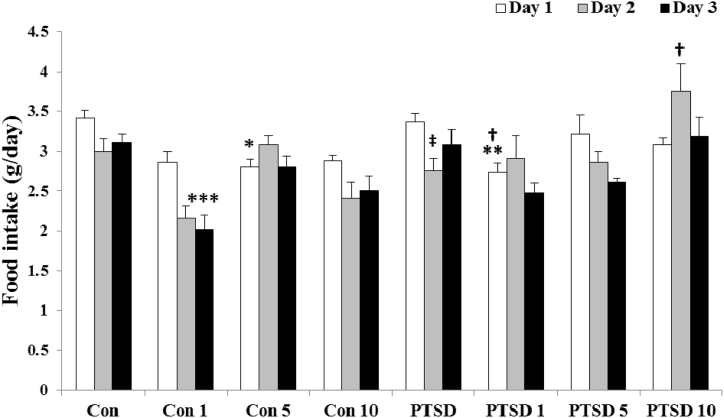
Illustrating the effects of different doses of Papaver rhoeas hydroalcoholic extract (1, 5 and 10 mg/kg) on food intake (g/day) of control and PTSD groups in three days of exposure to the stress box (control mice) or the predator stress (PTSD mice). All values are presented as mean ± SEM. *P < 0.05, **P < 0.01 and ***P < 0.001 as significant differences compared to control group. **†**P < 0.05 as significant differences compared to PTSD group. **‡**P < 0.05 as significant differences compared to day one for each group.

Fig. 3 illustrates food intake of mice during test. ANOVA test did not show a considerable change in food intake of the mice between different groups on the days 1, 2 and 3 [F (7, 40) = 3.956, F (7, 40) = 4.743 and F (7, 40) = 5.575, respectively]. Analysis of data at the different times for each group by repeated measures showed only a significant decrease in food intake of the PTSD mice on the second day (p < 0.018).

**Fig. 4:**
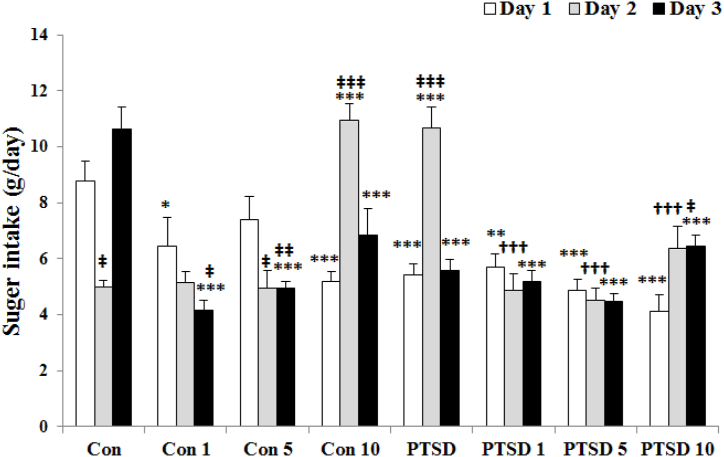
The effects of Papaver rhoeas hydroalcoholic extract (1, 5 and 10 mg/kg) on sugar intake (g/day) of control and PTSD groups during three days of exposure to the stress box (control mice) or the predator stress (PTSD mice). All values are presented as mean ± SEM. *P < 0.05, **P < 0.01 and ***P < 0.001 as significant differences compared to control group. **†††**P < 0.001 as significant differences compared to PTSD group. **‡**P < 0.05, **‡‡**P < 0.01 and **‡‡‡**P < 0.001 as significant differences compared to day one for each group.

The results of sugar intake during the test are shown at Fig. 4. Data analysis by ANOVA showed a decrease in the sugar intake levels in the mice of PTSD, treated PTSD and control groups on the first day [F (7, 40) = 9638]. Sugar intake of mice was lowest in all groups except PTSD and treated control mice with 10 mg/kg PR extract on the first day. On the third day [F (7, 40) = 15.092], analysis of data showed a low intake of sugar in the mice of all groups compared to control group like the first day. Analysis of data at the different times by repeated measures in each group showed that the sugar intake levels has a significant increase on the third and second days for control and PTSD mice, respectively. Whereas, administration of Papaver rhoeas extract decreased these increases in treated mice.

**Fig. 5:**
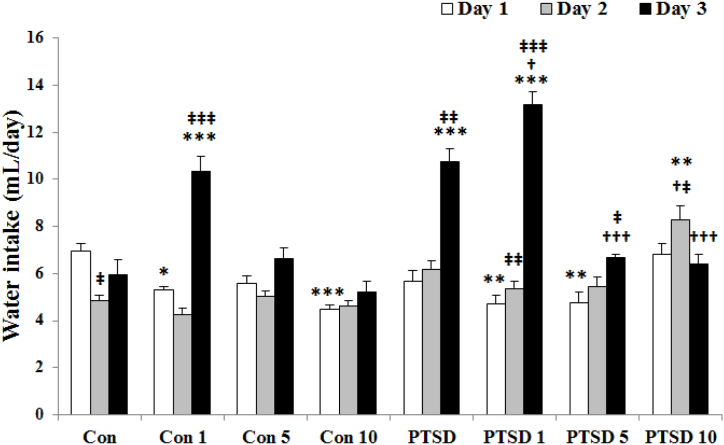
Illustrating the effects of different doses of Papaver rhoeas hydroalcoholic extract (1, 5 and 10 mg/kg) on water intake (mL/day) of control and PTSD groups during three days of exposure to the stress box (control mice) or the predator stress (PTSD mice). All values are presented as mean ± SEM. *P < 0.05, **P < 0.01 and ***P < 0.001 as significant differences compared to control group. **†**P < 0.05 and **†††**P < 0.001 as significant differences compared to PTSD group. **‡**P < 0.05, **‡‡**P < 0.01 and **‡‡‡**P < 0.001 as significant differences compared to day one for each group.

According to the results (Fig. 5), the most of treated control groups with the different doses of Papaver rhoeas extract as well as the treated PTSD mice with 1 & 5 mg Papaver rhoeas extract showed a decrease in water intake level on the first day, F (7, 40) = 6.853. On the second day, F (7, 40) = 12.809), only treated PTSD mice with 10 mg Papaver rhoeas extract showed the high levels of water intake. On the third day, F (7, 40) = 32.671), the mice of PTSD group as well as treated control and PTSD groups with 1 mg Papaver rhoeas extract showed the high levels of water intake. Also, analysis of data at the different times by repeated measures in each group showed that water intake considerably increased in PTSD mice as well as treated control and PTSD animals with 1 mg Papaver rhoeas extract on the third day compared to the first day.

### 3.2 Effect of Papaver rhoeas extract on the psychosocial behaviors

**Fig. 6:**
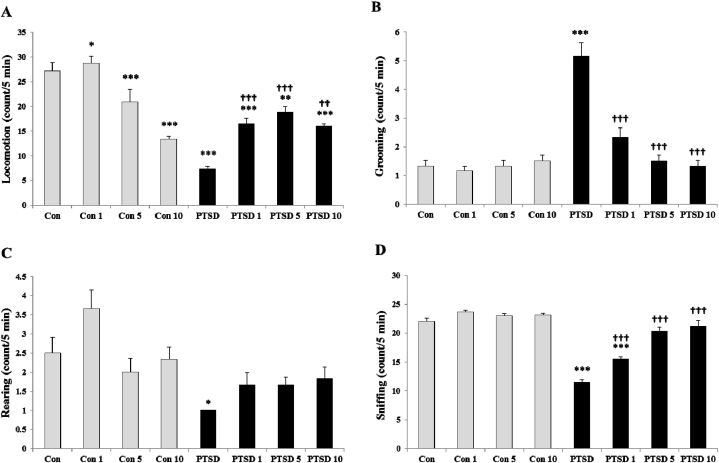
Effects of the different doses of Papaver rhoeas hydroalcoholic extract (1, 5 and 10 mg/kg) on the psychosocial behaviors (locomotion, grooming, rearing and sniffing) in the mice of control and PTSD groups at day 3 after exposing to the stress box (control mice) or predator stress (PTSD mice). All values are presented as mean ± SEM. *P < 0.05, **P < 0.01 and ***P < 0.001 as significant differences compared to control group. **††**P < 0.01 and **†††**P < 0.001 as significant differences compared to PTSD group.

Fig. 6 (A-D) illustrates the psychosocial behaviors (locomotion, grooming, rearing and sniffing) of the mice in the control and PTSD groups on the third day following exposing the animals to the stress box with presence and absent of the predator stress, respectively.

PTSD induction in the mice by exposing them to the predator stress significantly decreased the locomotor activity of animals [Fig. 6-A, F (7, 40) = 25.648, P < 0.001]. Also, the locomotor activity of animals significantly altered with administration of Papaver rhoeas extract both in control and PTSD mice. Papaver rhoeas extract at the doses of 5 and 10 mg/kg significantly decreased the locomotion of control mice, whereas 1 mg/kg of Papaver rhoeas extract increased it. On the other hand, treatment with different doses of Papaver rhoeas extract increased the locomotor activity of the PTSD mice.

Induction of PTSD in the mice by exposure of them to the predator stress significantly increased the grooming behavior [Fig. 6-B, F (7, 40) = 24.101, P < 0.001]. Treatment with Papaver rhoeas extract at the different doses significantly decreased the grooming behavior of PTSD mice (P < 0.001). However, administration of the different doses of Papaver rhoeas extract did not change the grooming behavior of the control mice (control groups).

Rearing behavior in the mice of control groups did not affect by the different doses of Papaver rhoeas extract administration (Fig. 6-C). PTSD induction, significantly decreased the rearing behavior in the PTSD mice compared to control mice F (7, 40) = 5.370, P < 0.05). Administration of the different doses of Papaver rhoeas extract briefly improved rearing behavior in the PTSD mice.

PTSD induction in the mice by exposing them to the predator stress significantly decreased the sniffing behavior of the animals (Fig. 6-D, F (7, 40) = 55.663, P < 0.001). Sniffing behavior did not alter by administration of Papaver rhoeas extract at the different doses in control mice. Whereas, treatment with Papaver rhoeas extract at the different doses significantly increased the sniffing behavior of PTSD mice in a dose-response manner.

### 3.3 Effect of Papaver rhoeas extract on the blood concentrations of corticosterone

**Fig. 7:**
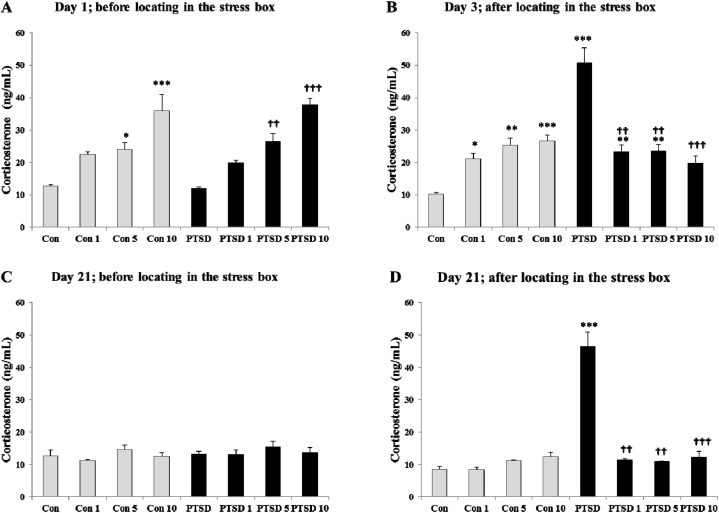
Effects of the different doses of Papaver rhoeas hydroalcoholic extract (1, 5 and 10 mg/kg) on the blood corticosterone concentrations in the mice of control and PTSD groups at day 1 (A); before exposure to the stress box (control groups) or predator stress (PTSD groups), day 3 (B); after exposure to the stress box (control groups) or predator stress (PTSD groups), day 21; before locating in the stress box (C) and day 21; after locating in the stress box (D). All values are presented as mean ± SEM. *P < 0.05, **P < 0.01 and ***P < 0.001 as significant differences compared to control group. **†**P < 0.05, **††**P < 0.01 and **†††**P < 0.001 as significant differences compared to PTSD group.

Fig. 7 (A-D) illustrates the blood concentrations of corticosterone in control and PTSD mice on the first day before exposing to the predator stress (7-A), third day after exposing to the predator stress (7-B), 21st day before locating in the stress box (7-C), and 21st day after locating in the stress box (7-D). On the first day before exposure to stress F (7, 40) = 16.789, the mean value of blood corticosterone for control and PTSD groups were 12.75 ± 0.39 ng/mL and 11.96 ± 0.49 ng/mL, respectively. Treatment with Papaver rhoeas hydroalcoholic extract increased the blood corticosterone levels in both treatment (control and PTSD) groups in a dose-response manner.

Three days after exposure to the predator stress F (7, 40) = 22.754, the mean value of blood corticosterone significantly increased in untreated PTSD group compared to untreated control group (50.78 ± 4.61, P < 0.001), whereas treatment with Papaver rhoeas hydroalcoholic extract reduced the blood levels of corticosterone in the treated PTSD groups near to value of blood corticosterone in the untreated control mice. Alterations of the blood corticosterone concentrations in control groups after locating in the stress box without stressor stimulus (lack of the predator) on the third day were like to on the first day before locating in the stress box.

On the 21st day before locating the mice in the stress box F (7, 40) = 0.838, the mean value of blood corticosterone was in the normal range for all animals in control and PTSD groups without any significant difference between them. Locating the mice in the stress box significantly increased the blood levels of corticosterone in PTSD group compared to control group F (7, 40) = 47.293, whereas treatment with Papaver rhoeas hydroalcoholic extract (at the doses of 1, 5 and 10 mg/kg) prevented the increase of blood corticosterone concentrations in the treated PTSD mice compared to untreated PTSD animals (p < 0.001).

### 3.4 Correlation between the psychosocial behaviors and blood corticosterone concentrations

**Fig. 8:**
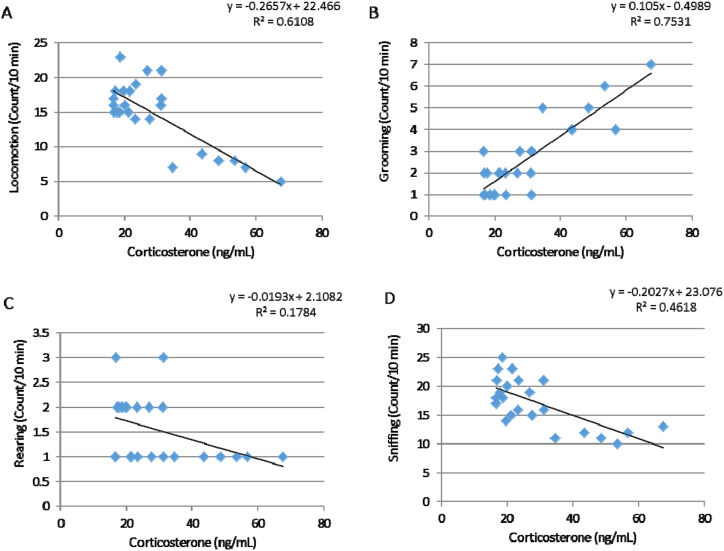
Correlation between the psychosocial behaviors and plasma corticosterone concentrations on the third days of the test in the PTSD groups. There is a high correlation between the grooming behavior and corticosterone concentration (B; R = 0.7531). Also, there is a modest correlation between the locomotion and sniffing behaviors with corticosterone concentrations (A and D; R = 0.6108 and R = 0.4618, respectively). Correlation was significant at the 0.05 level.

Pearson correlation was used to obtain correlation between the psychosocial behaviors and blood corticosterone concentrations of PTSD mice on the third day of the test (Fig. 8, A-D). The level confidence interval was 95%. There was a high correlation between the grooming behavior and corticosterone concentrations in PTSD mice on the third day (R = 0.7531) and correlation was significant at the 0.05 level. Also, there was a modest correlation between the locomotion and sniffing behaviors with corticosterone concentrations in PTSD mice on the third day (R = 0.6108 and R = 0.4618, respectively) and correlation was significant at the 0.05 level for them.

## 4 Discussion

According to previous studies, chronically induced PTSD in the rodents modified the functions and morphology of adrenal gland [10,26,27]. This gland plays a crucial role in response to the various psychophysiological situations to keep homeostatic regulation [12,28]. The findings of present study revealed that PTSD induction in the mice by exposing them to a hungry predator (a starved cat that habited to eat mouse) altered the adrenal functions and elevated the blood levels of corticosterone (Fig. 7). PTSD also changed the behavioral (psychosocial) and physiological responses of mice (Fig. 6). These altered behaviors, as confirmation of PTSD development, were observed on the 21st day of test when the animals were placed to the stress box without exposing to the hungry predator. On the other hand, treatment with Papaver rhoeas hydroalcoholic extract prevented the increment of blood corticosterone concentrations on the third day after exposure to the hungry predator and 21st day after exposure to the stress box. Also, Papaver rhoeas extract improved the behavioral and physiological responses of the PTSD mice. However, Papaver rhoeas extract increased blood corticosterone in a dose-response manner in control animals at the beginning of the study.

Experience of a severe stress makes an extensive change in the neuro-hormonal responses particularly in activity of the HPA axis [28,29]. Activation of this axis and release of cortisol from adrenal gland plays a key role in adaption response against stress [30]. Therefore, suitable response to stress requires to appropriate function of the cortisol signaling pathways [12,31]. Based on clinical and experimental evidence, there is a close relationship between the plasma cortisol levels and incidence of psychiatric disorders [11,32]. The results of the present study indicated that induction of PTSD by the repeated exposure of mice to a hungry predator like cat could increase the concentrations of blood corticosterone by 5–6 times following the last exposure. Accordingly, Starcevic et al. demonstrated that induction of PTSD by the predator exposure models increased cortisol levels after exposure to the psychosocial stress paradigm [13]. Also, Zoladz et al. reported that an acute psychosocial stress significantly increased the plasma cortisol levels of psychosocially stressed rats immediately after exposure to a predatory cat [33]. Although increased feedback inhibition of the HPA axis has been classically identified in chronic stress, hyperactivity of this axis is another relevant finding that observed following acute stress [34]. In general, these results propose the hypothesis that the behavioral responses to stress are regulated in stress-related brain circuits through chronic HPA axis dysregulation [12]. Based on the previous findings, the animal's heart rate and blood pressure also increased following the psychosocial stress [33]. Therefore, an increase in the blood corticosterone concentrations after the psychosocial stressors like exposure of mice to a predator cat is consistent with the findings from PTSD patients who show high cortisol levels in anticipation of anxiety-provoking conditions and in response to trauma-related stimuli [14,35]. Although people suffering from PTSD are characterized by the high concentrations of corticotropin-releasing hormone (CRH), the basal cortisol level decreases due to the enhanced negative feedback suppression of the HPA axis [2,36]. In this regard, decreased the blood cortisol levels and damage to the glucocorticoid signaling pathways have been demonstrated in patients with psychiatric disorders [36,37].

PTSD is a debilitating psychiatric condition that developed after experience of a stressful or traumatic event in the people life [1,6]. The psychological symptoms of this disorder can persist for a long time and appears under certain conditions [2,38]. In this regard, activation of the HPA axis and followed increased blood cortisol is one of the common responses to stress in animals, when exposed to a hungry predator to induce PTSD [10,33]. According to our results, an increase in the blood corticosterone levels by 5–6 times was observed on the third day after induction of PTSD (or after three consecutive days of exposure to the hungry predator). If PTSD had been developed in the animal, an increase in HPA axis and followed increased blood corticosterone might have been observed in response to exposing the animals to the stress box even without the presence of the predator. The results of blood corticosterone alterations on the 21st day well confirmed the PTSD induction in the animals, because exposure to the stress box without the presence of the hungry predator was able to increase the blood corticosterone concentrations up to 5–6 times similar to the third day after PTSD induction. Based on previous studies, the behavioral and neurobiological responses to fear arise from a pattern associated with a Pavlovian learning process called fear conditioning [5]. In this process, when a neutral stimulus is paired with an unconditioned stimulus (like a shock) the relationship between the two stimuli is learned [39]. During in this type of learning, the subject presents a response that is named the conditioned response [5]. The persistent alterations in HPA function and HPA axis hypersensitivity are other relevant findings in PTSD [28,40]. Interestingly, HPA axis is affected chronically by the early life stress in the animal models and human studies [4,9]. Furthermore, increased the blood adrenocorticotropic hormone (ACTH) levels as well as cortisol secretion from adrenal gland in response to the psychosocial stressors has been observed in the adult women who were abused during childhood [9,40]. Another relevant finding in HPA hyperactivity in PTSD patients is higher levels of CRH in the cerebrospinal fluid [41,42]. Furthermore, elevated CRH levels are connected to appearance of several psychosocial behaviors in PTSD patients, including anxiety, fear conditioning and the startle response [34,43]. Hence, it is suggested that decreased locomotor activity of animals in the present study as well as alterations of the psychosocial behaviors (such as sniffing, rearing and grooming) in PTSD mice might be associated to the altered HPA function (Fig. 8). The results of PTSD-related psychosocial behaviors like locomotion, grooming, rearing and sniffing in the present study are consistent with other studies. According to the findings of Mendes-Gomes et al., defensive immobility increased following repeated exposure of the mice to a snake as an experimental model of PTSD [44]. Their findings also confirmed our psychosocial behaviors following PTSD induction in mice. In this regard repeated exposure of the mice to a snake for making PTSD increased grooming behavior and decreased rearing response [44]. McGuire et al., also reported that posttraumatic stress-like behaviors were observed in rats following recovery from exposing repeated trauma [45]. In this regard, grooming behavior increased in these animals but rearing response unchanged. Moreover, PTSD induction in rats decreased locomotion and rearing behavior, but increased bouts of grooming and instances of immobility [46]. Furthermore, evidence from animal and human studies showed the events linked to PTSD modify the neural circuits and neurotransmitter systems of brain regions that are crucial for regulation of the psychosocial behaviors such as cognition and affective behavior [4,9,27]. These regions are included the hippocampus, the HPA axis, neural networks of fear responses and cognition as well as the dopamine and serotonin (5-HT) systems [9].

The results of present study revealed that treatment with Papaver rhoeas hydroalcoholic extract increased the blood corticosterone levels, in a dose-response manner, in control animals without receiving any stress (Fig. 7-A). These findings have been demonstrated by the previous studies [25,47]. Mirzaei et al. demonstrated that orally administration of Papaver rhoeas hydroalcoholic extract significantly increased blood corticosterone in mice both in normal condition and under stress induced by an electrical shock [47]. Osanloo et al. also, reported that the different doses of Papaver rhoeas hydroalcoholic extract (1, 10, 30 and 100 mg/kg) increased the blood corticosterone levels in male and female mice in a dose-independent manner in a force swimming model of depression [25]. In this regard, the findings of previous studies suggested that Papaver rhoeas extract increased the concentrations of blood cortisol probably by the peripheral effects through stimulation of adrenal gland [48,49]. On the other hand, Papaver rhoeas extract decreased the blood corticosterone levels in treated PTSD mice compared to treated control animals (Fig. 7-B). It also prevented the increase of blood corticosterone in treated PTSD mice that exposed to the stress box on the 21st day (Fig. 7-B). Induction of acute stress in rodent increases corticosterone secretion through activation of the HPA pathway [12,34]. Hence, it is possible that Papaver rhoeas extract has decreased the blood corticosterone concentrations in the PTSD mice against stress through inhibition of HPA axis. This effect is in contrast to the peripheral effects of Papaver rhoeas extract, which stimulates corticosterone secretion from adrenal gland. This claim has not been confirmed by the research and more studies are needed to clarify it. However, the results of previous studies have indicated that the Papaver rhoeas extract contains several components such as papauric acid, papaverine and muconic acid that they could affect the release of different brain neurotransmitters [18,21,22]. The *anti*-glutamate effect of Papaver rhoeas extract is another relevant finding in the experimental studies that suggested for reduction of the depression symptoms [24,25]. Based on previous findings, endogenous glutamate and its receptor subtypes are important in triggering HPA axis, and then, increased hypothalamic NMDA receptor activity participates in HPA hyperactivity during chronic stress [50,51]. Since simultaneous inhibition of the glutamate receptors (NMDA and AMPA) leads to reduction of the plasma ACTH levels in response to the different stress stimuli, Papaver rhoeas extract might decrease the blood corticosterone concentrations in PTSD mice by glutamate receptor blockage in brain [52]. Another relevant finding is the anti-dopaminergic effects of Papaver rhoeas alkaloids that they could reduce the activity of HPA axis under stress [23]. As dopamine increases the excitability of HPA axis under stress through both D1 and D2 receptors, using dopamine receptors (D1R and D2R) antagonists could diminish the activity of HPA axis [53]. According our results, inhibition of HPA axis and decreased blood corticosterone by Papaver rhoeas extract might be involved in improvement of the psychosocial behaviors in PTSD mice. Accordingly, Papaver rhoeas extract improved the depression symptoms and increased locomotor activity of mice by inhibition of corticosterone secretion [25].

According to our results, induction of PTSD in the mice by predator exposure caused a body weight reduction during the test. These mice showed a significant reduction in food intake particularly on the second day, but sugar and water intake increased in these animals. Numerous reports have demonstrated that exposure to chronic stress can promote either obesity or anorexia within certain dietary environments [54,55]. Previously, stress-induced anoroxia has been demonstrated by a large number of the studies in the rodents [56,57]. Petrovich et al.,. using a more intense and imminent psychological stress in the rat models such as conditioned fear demonstrated that intense and imminent threat promoted hypophagia and anorexia as well as decreased food intake [58]. In this regard, central amigdal (CeA), likely working with the ventromedial prefrontal cortex (vmPFC) and lateral hypothalamic area (LHA), plays as an essential role for reducing food intake during fear anticipation in rats that is associated with CRH-driven hyperactivity of HPA axis [56]. Hence, in the present study the PTSD mice showed a significant reduction in food intake on the second day. Accordingly, some types of anorexia are fulfilled by the criteria of cortisol hypersecretion that are associated with an increased plasma cortisol and urinary cortisol excretion [59,60]. Progressive experimental evidence demonstrated that stress could either increase or decrease caloric intake [56]. Comparable changes in palatable food intake in the rodents have been reported during stress [61]. When these rodents exposed to chronic stress in accompany with presentation of both highly-palatable food (like sucrose or sugar) and lessly-palatable food (normal rat chow), they preferred to eat a large proportion of their daily calories as the highly-palatable food [56,62,63]. These findings confirmed our results because the PTSD mice showed a decrease in food intake and a increase in sugar and water intake. Moreover, the findings from eperimental studies powerfully propose that elevated palatable food intake in rodents is associated with reduced stress indices through unknown mechanism [56,63].

On the other hand, treated PTSD mice by the different doses of Papaver rhoeas extract increased body weight of animals particularly in higher doses. Treatment with Papaver rhoeas extract also briefly improved food intake and sugar intake during test. According to previous findngs, activation of HPA axis as well as increased plasma cortisol has a key role in anorexia development and reduction of food intake under stress condition [54,59]. A relevant finding in the present study was HPA axis inhibition by Papaver rhoeas extract that confirmed by decreased blood corticosterone. Hence, it is suggested that improving food intake and bodey weight by Papaver rhoeas extract might be linked to decreased blood corticosterone. According to the findings, Papaver rhoeas extract also influences the release of several neurotrasmitters in rat brain that might affect anorexia and food intake [24,25]. Hyperactivation of HPA axis by excessive excitation via glutamate in PTSD condition can influence anorexia and food intake [55]. Therefore, the *anti*-glutamate effects of Papaver rhoeas extract might be another relevant mechanism in the PTSD mice that is suggested for improvement of food intake and body weight [24,25]. The mice of control and treated control groups (at doses of 1 & 5 mg/kg) showed an increase in body weight during the test. Whereas, treated mice with higher doses of Papaver rhoeas extract showed a decrease in body weight. These results might be developed in these animals due to a significant reduction in food intake as well as an increased sugar and water intake particularly on the second day. Additionally, Papaver rhoeas extract increased blood corticosterone probably via HPA axis that might be important for changing rat food from a lessly-palatable food (normal rat chow) to a highly-palatable food (sugar), [56]. These altrations might be the main reasons of body weight reduction in treated control mice with higher doses of Papaver rhoeas extract.

## 5 Conclusion

According to our results, Papaver rhoeas extract probably prevents appearance of the psychosocial behaviors as well as alterations of HPA axis and physiological variables in the experimental model of PTSD. Papaver rhoeas extract decreased blood corticosterone concentrations in PTSD mice, and prevented alterations of blood corticosterone in PTSD mice when exposed to the stress stimuli. Accordingly, inhibition of HPA axis and decreased blood corticosterone by Papaver rhoeas extract might be involved in improvement of the physiological and psychosocial behaviors in PTSD mice.

## Author contribution statement

Shima Shahyad: Conceived and designed the experiments; Performed the experiments; Contributed reagents, materials, analysis tools or data; Wrote the paper.

Hedayat Sahraei: Conceived and designed the experiments; Performed the experiments; Analyzed and interpreted the data; Wrote the paper.

Kamal Mousallo: Conceived and designed the experiments; Analyzed and interpreted the data.

Gila Pirzad Jahromi: Conceived and designed the experiments; Wrote the paper.

Mohammad Taghi Mohammadi: Conceived and designed the experiments; Contributed reagents, materials, analysis tools or data; Wrote the paper.

## Data availability statement

Data included in article/supp. material/referenced in article.

## Declaration of competing interest

The authors declare that there is no conflict of interest.
